# Application of machine learning in the prediction of deficient mismatch repair in patients with colorectal cancer based on routine preoperative characterization

**DOI:** 10.3389/fonc.2022.1049305

**Published:** 2022-12-22

**Authors:** Dong Xu, Rujie Chen, Yu Jiang, Shuai Wang, Zhiyu Liu, Xihao Chen, Xiaoyan Fan, Jun Zhu, Jipeng Li

**Affiliations:** ^1^ Division of Digestive Surgery, Xijing Hospital of Digestive Diseases, Air force Medical University, Xi’an, China; ^2^ School of Clinical Medicine, Xi’an Medical University, Xi’an, China; ^3^ Department of Neurosurgery, Xijing Hospital, Air Force Medical University, Xi’an, China; ^4^ State Key Laboratory of Cancer Biology, Institute of Digestive Diseases, Xijing Hospital, The Fourth Military Medical University, Xi’an, China; ^5^ Xi’an Institute of Flight of the Air Force, Ming Gang Station Hospital, Minggang, China; ^6^ Department of General Surgery, The Southern Theater Air Force Hospital, Guangzhou, China; ^7^ Department of Experiment Surgery, Xijing Hospital, Fourth Military Medical University, Xi’an, China

**Keywords:** colorectal cancer, deficient mismatch repair, real-world research, machine learning, routine preoperative characterization

## Abstract

**Simple summary:**

Detecting deficient mismatch repair (dMMR) in patients with colorectal cancer is essential for clinical decision-making, including evaluation of prognosis, guidance of adjuvant chemotherapy and immunotherapy, and primary screening for Lynch syndrome. However, outside of tertiary care centers, existing detection methods are not widely disseminated and highly depend on the experienced pathologist. Therefore, it is of great clinical significance to develop a broadly accessible and low-cost tool for dMMR prediction, particularly prior to surgery. In this study, we developed a convenient and reliable model for predicting dMMR status in CRC patients on routine preoperative characterization utilizing multiple machine learning algorithms. This model will work as an automated screening tool for identifying patients suitable for mismatch repair testing and consequently for improving the detection rate of dMMR, while reducing unnecessary labor and cost in patients with proficient mismatch repair.

**Background:**

Deficient mismatch repair (dMMR) indicates a sustained anti-tumor immune response and has a favorable prognosis in patients with colorectal cancer (CRC). Although all CRC patients are recommended to undergo dMMR testing after surgery, current diagnostic approaches are not available for all country hospitals and patients. Therefore, efficient and low-cost predictive models for dMMR, especially for preoperative evaluations, are warranted.

**Methods:**

A large scale of 5596 CRC patients who underwent surgical resection and mismatch repair testing were enrolled and randomly divided into training and validation cohorts. The clinical features exploited for predicting dMMR comprised the demographic characteristics, preoperative laboratory data, and tumor burden information. Machine learning (ML) methods involving eight basic algorithms, ensemble learning methods, and fusion algorithms were adopted with 10-fold cross-validation, and their performance was evaluated based on the area under the receiver operating characteristic curve (AUC) and calibration curves. The clinical net benefits were assessed using a decision curve analysis (DCA), and a nomogram was developed to facilitate model clinical practicality.

**Results:**

All models achieved an AUC of nearly 0.80 in the validation cohort, with the stacking model exhibiting the best performance (AUC = 0.832). Logistical DCA revealed that the stacking model yielded more clinical net benefits than the conventional regression models. In the subgroup analysis, the stacking model also predicted dMMR regardless of the clinical stage. The nomogram showed a favorable consistence with the actual outcome in the calibration curve.

**Conclusion:**

With the aid of ML algorithms, we developed a novel and robust model for predicting dMMR in CRC patients with satisfactory discriminative performance and designed a user-friendly and convenient nomogram.

## Introduction

Colorectal cancer (CRC) is the third most common cancer and the second most common cause of cancer-related death worldwide, posing an ongoing threat to public health (1). As a molecular subtype of CRC, microsatellite instability (MSI) or deficient mismatch repair (dMMR) plays a prominent role in the formation of tumors and development of cancer and is therefore a potential therapeutic target (2). dMMR leads to the accumulation of multiple mutations and MSI to further induces tumorigenesis (3), which is observed in approximately 15% of CRC cases and incorporated in the universal screening protocol for Lynch syndrome (4).

MSI or dMMR has a favorable prognosis but gains no benefit from neoadjuvant chemotherapy with fluorouracil in CRC patients with stage II (5). More importantly, recent studies elucidate that immune checkpoint blockade (ICB) treatment in patients with MSI or dMMR can yield remarkable clinical benefits (6). Further investigations suggest that the benefit of ICB treatment in these patients is not limited to CRC but encompasses all solid tumors (7). Now, MSI or dMMR has been approved by the FDA as the first pan-cancer biomarker for immunotherapy response (8). Given the extensive clinical applications, MSI or dMMR testing has been recommended by multiple international guidelines for all CRC patients (9–11). However, in clinical practice, not all CRC patients are tested for MSI or dMMR, especially those in developing cities and hospitals; this is because testing requires particular genetic or immunohistochemical examinations, which are costly and time-consuming and rely on excellent pathology laboratories and doctors (12). Therefore, a low-cost tool that can be used in all CRC patients from different cities and hospitals is essential.

Machine learning (ML) has shown great potential in identifying features of disease subtypes and outcomes, with successful application in disease screening (13), prognosis evaluation (14), and efficacy prediction (15). Previous studies have suggested that ML can recognize pathomorphological characteristics that contribute to MSI or dMMR from hematoxylin and eosin-stained tissue sections. Although these results are promising, the performance of ML is poorer when tissue areas are smaller than surgically resected specimens and is subject to inter-hospital variability in relation to the quality of histological sections (16, 17). Meanwhile, researchers have discovered susceptibility genes associated with MSI or dMMR from genomic sequencing data based on ML algorithms. However, genetic testing is expensive, making it difficult to test all patients (18).

In this study, we aimed to develop an ML-based model for predicting dMMR that is easily accessible and consistent among different regions and hospitals based on routine clinical data. As an automated screening tool, the model is expected to complement further confirmatory testing, while reducing unnecessary labor and cost in patients with proficient mismatch repair (pMMR).

## Methods

### Patients

This retrospective study included a primary cohort of CRC patients who underwent surgical resection and dMMR testing between January 2015 and August 2021 in the Xijing Hospital of Digestive Diseases, Air Force Military Medical University (Shaanxi, China). The inclusion criteria were as follows: (1) primary colorectal cancer confirmed *via* cytological and histological examinations; (2) dMMR testing; and (3) complete clinical and pathological data. In contrast, the exclusion criteria were as follows: (1) coexistence of other primary malignant tumors and (2) chemoradiotherapy, immunotherapy, and other related anti-tumor therapies before surgery. In total, 5596 patients and 80 clinical features, including primary demographic characteristics, tumor information, and routine laboratory data, were investigated in our study. All data retrieved from electronic patient records were further transformed and normalized to facilitate feature selection and model building, and all clinical data were explicitly scrutinized. The study was approved by the Medical Ethics Committee of the First Affiliated Hospital of the Air Force Military Medical University (No. KY20112170-C-1).

### Model development

Traditional models depend largely on human-selected features, while ML can learn features from data, which allows researchers to obtain untapped information and detect difficult-to-discern patterns (19). Therefore, we constructed a predictive model based on ML. In this study, the cohort was split into separate training and validation sets at an 8:2 ratio using the light gradient boosting machine (LGBM) (random state = 3), which could maintain the same ratio of positive-to-negative samples in the training and validation sets. As a classical approach, cross-validation is typically exploited to avoid overfitting of training data in ML (20). Thus, in the training set, we employed k-fold cross-validation (k = 10), a procedure that uses subsets of data to iteratively train and validate the predictive performance of a model. In this procedure, a cohort is randomly divided into 10 subsets, of which nine subsets are included in the training set and the remaining subset in the testing set; the optimal hyperparameters arise from the combination of the best cross-validation results and the model that will provide the best predictive performance on a new sample (21). For model development, we trained eight basic models based on eight different ML pipelines. The LGBM is a highly efficient gradient boosting decision tree suitable for scenarios with large amounts of data and high-dimensional features (22). Random forest (RF) is an ensemble learning algorithm that builds and merges diverse decision trees to obtain the optimal classification performance (23). Gaussian Naive Bayesian (GNB), a supervised learning method, approaches the classification task with the naive assumption of independence between every pair of features (24). The K-nearest neighbor (KNN) is a simple and effective classification algorithm using the k-nearest points of inputs to predict responses (25). The multilayer perceptron (MLP) is a feedforward neuronal network consisting of an input layer, an output layer, and one or more hidden layers that are closely connected; it is widely used in distinguishing data that are not linearly separable (26, 27). A classification and regression tree (CART) is a modeling approach for classification (binary response) and regression (continuous response) that has been successfully utilized in clinical practice (28). The support vector machine (SVM) is currently the mainstream classifier in ML, which has been intensively applied for pattern recognition and data classification (29). Logistic regression (LR) is a generalized linear model used to solve binary classification problems, which can fit binary or multinomial LR (30). To further optimize the model, we applied bagging bootstrap aggregation, an ensemble technique used for classification or regression, to determine the hyperparameters of the basic models (31). Next, we built a fusion model with hard voting (based on the average of the bagging model) or soft voting (based on the weighted average of the predicted class probabilities) (32).

### Feature importance

Shapley additive explanation (SHAP), one of the most optimal ML explication methods based on the game theory, allows both local and global interpretabilities of the output of any ML-based model (33). To determine which features contributed most to the model predictions, we calculated and visualized the SHAP values on the stacking predictions. Each SHAP value measured how much each feature contributed, either positively or negatively, to the risk of dMMR assigned by the model.

### Model performance

We adopted the area under the receiver operating characteristic curve (AUC) as the mainstay parameter of model performance. Furthermore, we calculated and compared the sensitivity, specificity, precision, negative predictive value (NPV), false discovery rate (FDR), accuracy, average precision (AP), and confusion matrix to further assess the comprehensive performance of the ML-based models. In addition, a decision curve analysis (DCA) was employed to evaluate the clinical net benefit of the models (34).

### Statistical analysis

Statistical analysis was conducted using R (version 4.1.2; https://www.Rproject.org), and ML modeling was performed using Python (version 3.6.5; https://www.python.org). The following Python packages were used: “imblearn,” “sklearn,” “lightgbm,” “randomForest,” and “mlxtend.” Meanwhile, the following R packages were utilized: “tableone,” “survival,” “mice,” and “rms.” Qualitative data were compared between the two groups using the χ^2^ test or Fisher’s exact test. P < 0.05 was considered statistically significant.

## Results

### Baseline clinical data

**Figure 1:**
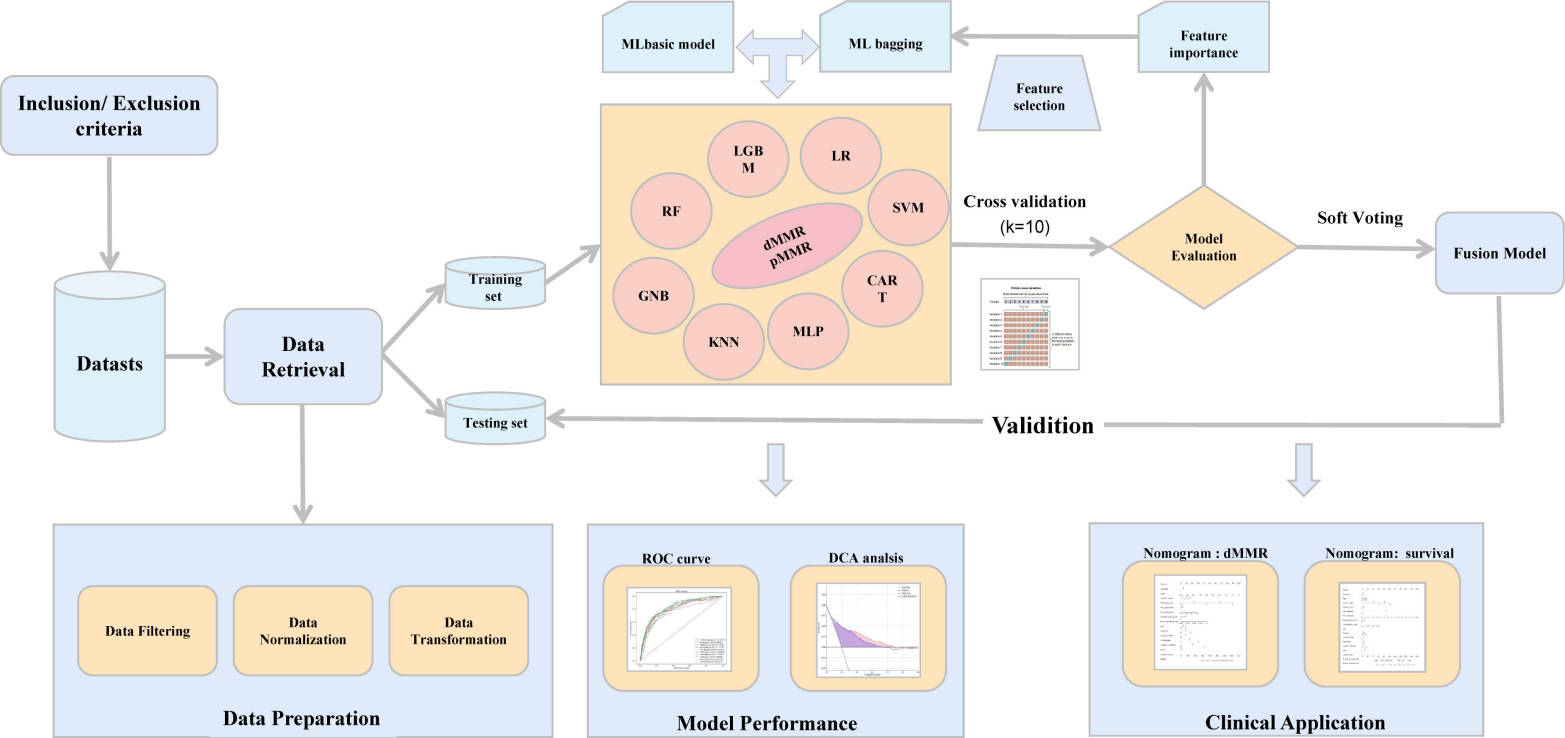
The workflow of the study. ML, machine learning; ROC, receiver operating characteristic; DCA, decision curve analysis.

The flowchart of this study is shown in **Figure 1**. The primary data were extracted from electronic medical records between January 2015 and August 2021. According to the inclusion and exclusion criteria, 5596 patients were enrolled as the study population: 4476 patients included in the training cohort and 1120 in the validation cohort. No significant differences were found in the prevalence of dMMR between the two cohorts. dMMR was found in 508 (11.3%) and 110 (9.82%) patients in the training and validation cohorts, respectively. *There* was *no* significant difference between *the* difference of clinical characteristics between *the training* cohorts *and* testing cohorts, including gender, age, tumor type, primary site, histological grade, tumor size and other experimental indicators (P >0.05). The baseline characteristics of the cohorts are shown in **Table 1**.

**Table 1:** Characteristics of patients in the training and validation cohorts.

	Training Cohort	Validation Cohort	
Characteristic	(n=4476)	(n=1120)	P value
Gender, No (%)			0.975
Male	2614 (58.4%)	671 (59.9%)	
Female	1862 (41.6%)	449 (40.1%)	
Age, mean ± SD, years	60.49±12.37	60.32±12.54	0.995
Tumor type			0.995
Common adenocarcinoma	3796 (84.81%)	965 (86.16%)	
Adenosquamous carcinoma	1 (0.02%)	0 (0%)	
Mucinous adenocarcinoma	626 (13.99%)	139 (12.41%)	
Squamous cell carcinoma	6 (0.13%)	2 (0.18%)	
Neuroendocrine carcinoma	28 (0.63%)	9 (0.80%)	
Signet ring cell carcinoma	19 (0.42%)	5 (0.45%)	
Primary site			0.995
Cecum-ascending colon	855 (19.10%)	197 (17.59%)	
Transverse colon	161 (3.60%)	35 (3.13%)	
Descending colon	239 (5.34%)	67 (5.98%)	
Sigmoid colon	770 (17.20%)	209 (18.66%)	
Rectum	2451 (54.76%)	612 (54.64%)	
Albumin level, No (%), (g/L)			0.995
<30	38 (0.85%)	10 (0.89%)	
≥30	4438 (99.15%)	1110 (99.11%)	
Globulin level, (g/L) median (interquartile range)	27.4 (24.6,30.3)	27.45 (24.4,30.3)	0.995
Lymphocyte ratio, No (%)			0.99
<0.255	2225 (49.71%)	550 (49.11%)	
≥0.255	2251 (50.29%)	570 (50.89%)	
Eosinophils ratio, median (interquartile range)	0.01 (0.01,0.03)	0.01 (0.01,0.03)	0.975
HB level, No (%), (g/L)			0.995
< 30	0 (0%)	0 (0%)	
30-60	25 (0.56%)	5 (0.45%)	
60-90	389 (8.69%)	80 (7.14%)	
>90	4062 (90.75%)	1035 (92.41%)	
Platelet level, No (%), (×10^9^/L )			0.995
<100	89 (1.99%)	20 (1.79%)	
100-400	4116 (91.96%)	1051 (93.84%)	
>400	271 (6.05%)	49 (4.37%)	
CA125, No (%)			0.975
Normal	3967 (88.63%)	985 (87.95%)	
Abnormal	509 (11.37%)	135 (12.05%)	
Histological grade			0.995
Well differentiated	992 (22.16%)	243 (21.70%)	
Moderately differentiated	2807 (62.71%)	701 (62.59%)	
Poorly differentiated	677 (15.13%)	176 (15.71%)	
Tumor volume, No (%), (cm^3^)			0.995
<9	1472 (32.89%)	357 (31.87%)	
9-17.5	1517 (33.89%)	374 (33.39%)	
≥17.5	1487 (33.22%)	389 (34.74%)	
NLR, No (%)			0.995
<2.56	2236 (49.96%)	569 (50.80%)	
≥2.56	2240 (50.04%)	551 (49.20%)	

HB, hemoglobin; CA125, carbohydrate antigen 125; NLR, neutral lymphoid ratio

Fourteen clinical features with better performance were eventually employed to build the final model: age at diagnosis, sex, hemoglobin (HB) level, platelet count, albumin level, globulin level, lymphocyte ratio, eosinophil ratio, neutral lymphoid ratio (NLR), CA125 level, primary site, histologic tumor grade, tumor type, and tumor volume. The baseline characteristics of the dMMR and pMMR groups are shown in detail in **Table 2**.

**Table 2:** Comparison of clinical characteristics in the dMMR and pMMR group.

	Training dataset			Validation dataset		
Characteristic	dMMR (n=508)	pMMR (n=3968)	P value	dMMR (n=110)	pMMR (n=1010)	P value
Gender, No (%)			0.191			0.697
Male	283 (55.71%)	2331 (58.74%)		64 (58.18%)	607 (60.1%)	
Female	225 (44.29%)	1637 (41.26%)		46 (41.82%)	403 (39.9%)	
Age, mean ± SD, years	56.94±14.2	60.95±12.04	<0.001	55.9±14.03	60.8±12.28	0.001
Tumor type			<0.001			0.074
Common adenocarcinoma	365 (71.85%)	3431 (86.47%)		86 (78.18%)	879 (87.03%)	
Adenosquamous carcinoma	0 (0%)	1 (0.03%)		0 (0%)	0 (0%)	
Mucinous adenocarcinoma	135 (26.57%)	491 (12.37%)		23 (20.91%)	116 (11.49%)	
Squamous cell carcinoma	0 (0%)	6 (0.15%)		0 (0%)	2 (0.2%)	
Neuroendocrine carcinoma	5 (0.98%)	23 (0.58%)		1 (0.91%)	8 (0.79%)	
Signet ring cell carcinoma	3 (0.59%)	16 (0.4%)		0 (0%)	5 (0.5%)	
Primary site			<0.001			<0.001
Cecum-ascending colon	237 (46.65%)	618 (15.57%)		53 (48.18%)	144 (14.26%)	
Transverse colon	43 (8.46%)	118 (2.97%)		13 (11.82%)	22 (2.18%)	
Descending colon	71 (13.98%)	168 (4.23%)		17 (15.45%)	50 (4.95%)	
Sigmoid colon	59 (11.61%)	711 (17.92%)		12 (10.91%)	197 (19.5%)	
Rectum	98 (19.29%)	2353 (59.3%)		15 (13.64%)	597 (59.11%)	
Albumin level, No (%), (g/L)			<0.001			<0.001
<40	163 (32.09%)	653 (16.46%)		32 (29.09%)	159 (15.74%)	
≥40	345 (67.91%)	3315 (83.54%)		78 (70.91%)	851 (84.26%)	
Globulin level, (g/L) median (interquartile range)	27.9 (24.78,30.83)	27.4 (24.6,30.3)	0.021	27.85 (24.8,31.37)	27.4 (24.3,30.2)	0.129
Lymphocyte ratio, No (%)			<0.001			0.016
<0.255	305 (60.04%)	1920 (48.39%)		66 (60%)	484 (47.92%)	
≥0.255	203 (39.96%)	2048 (51.61%)		44 (40%)	526 (52.08%)	
Eosinophils ratio, median (interquartile range)	0.013 (0.007,0.023)	0.015 (0.008,0.025)	0.016	0.013 (0.006,0.023)	0.015 (0.008,0.026)	0.281
HB level, No (%), (g/L)			<0.001			<0.001
< 30	0 (0%)	0 (0%)		0 (0%)	0 (0%)	
30-60	8 (1.57%)	17 (0.43%)		0 (0%)	5 (0.5%)	
60-90	97 (19.09%)	292 (7.36%)		19 (17.27%)	61 (6.04%)	
>90	403 (79.33%)	3659 (92.21%)		91 (82.73%)	944 (93.47%)	
Platelet level, No (%), (×10^9^/L )			<0.001			0.004
<100	8 (1.57%)	81 (2.04%)		0 (0%)	20 (1.98%)	
100-400	421 (82.87%)	3695 (93.12%)		99 (90%)	952 (94.26%)	
>400	79 (15.55%)	192 (4.84%)		11 (10%)	38 (3.76%)	
CA125, No (%)			<0.001			0.003
Normal	400 (78.74%)	3567 (89.89%)		87 (79.09%)	898 (88.91%)	
Abnormal	108 (21.26%)	401 (10.11%)		23 (20.91%)	112 (11.09%)	
Histological grade			<0.001			0.002
Well differentiated	120 (23.62%)	872 (21.98%)		26 (23.64%)	217 (21.49%)	
Moderately differentiated	238 (46.85%)	2569 (64.74%)		55 (50%)	646 (63.96%)	
Poorly differentiated	150 (29.53%)	527 (13.28%)		29 (26.36%)	147 (14.55%)	
Tumor volume, No (%), (cm^3^)			<0.001			<0.001
<9	92 (18.11%)	1380 (34.78%)		22 (20%)	335 (33.17%)	
9-17.5	117 (23.03%)	1400 (35.28%)		19 (17.27%)	355 (35.15%)	
≥17.5	299 (58.86%)	1188 (29.94%)		69 (62.73%)	320 (31.68%)	
NLR, No (%)			<0.001			0.029
<2.56	204 (40.16%)	2032 (51.21%)		45 (40.91%)	524 (51.88%)	
≥2.56	304 (59.84%)	1936 (48.79%)		65 (59.09%)	486 (48.12%)	

dMMR, mismatch-repair deficiency; HB, hemoglobin; CA125, carbohydrate antigen 125; NLR, neutral lymphoid ratio.

### Parameters

We trained the LGBM with a depth of 5, a learning rate of 0.012, basic learners of 230, a leaf size of 8, and maximum bins of 256. For the RF and CART, the maximum depths of the basic trees were 10, and the basic learners were 500. For the KNN, the leaf size was 30, and the optimum number of neighbors was 400. For the MLP, we used three hidden layers with a size of 50, 30, and 10, respectively; a learning rate of 0.08; and the Adam optimizer and ReLU activation function. For the SVM, we combined a C value of 0.01 with a kernel smoothing parameter of 0.01. Each bagging model was also linked with 10 datasets generated by random sampling for 10 times, allowing the construction of the predictive models based on identical basic algorithms and evaluation of their performance using the 10-fold cross-validation test. The eventual stacking model consisted of eight bagging models, with final weight values of 42 (LGBM), 12 (RF), 2 (GNB), 4 (KNN), 4 (MLP), 10 (DT), 34 (SVM), and 24 (LR).

### Feature importance

**Figure 2:**
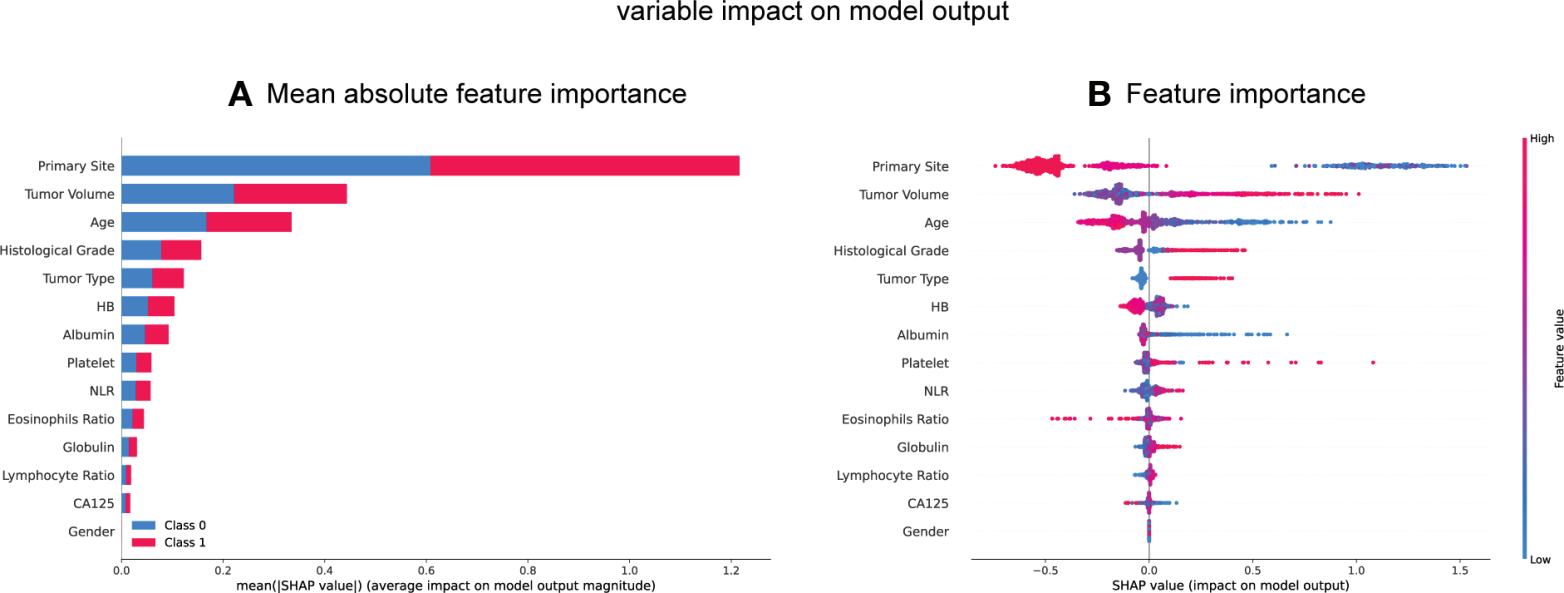
SHAP summary plot of the 14 feature of the stacking model. **(A, B)** Each dot corresponds to each feature attribution value for the model of each patient, with positive values indicating a contribution that increase the probability of dMMR development while negative values indicating a contribution that decreases the probability. Dots are colored according to the values of features for the respective patient and accumulate vertically to depict density. Red represents higher feature values, and blue respective lower feature values. HB, hemoglobin; NLR, neutral lymphoid ratio; CA125, carbohydrate antigen 125.

To further determine the association of each feature with the outcome of mismatch repair (MMR), we employed the SHAP values. Meanwhile, we calculated and visualized the significance of each feature by analyzing the SHAP values in all individual models (**Supplementary Table S1**) and stacking model (**Figure 2**). The 10 most important features in the stacking model were the primary tumor site, tumor volume, age at diagnosis, histological tumor grade, tumor type, and preoperative HB level, albumin level, platelet count, NLR, and eosinophil ratio. Notably, a larger tumor volume, younger age, higher histological tumor grade, higher platelet count, and higher NLR ratio were associated with a higher risk of dMMR, while a higher preoperative HB level, albumin level, and eosinophil ratio were related to a lower risk of dMMR.

### Model performance

**Figure 3:**
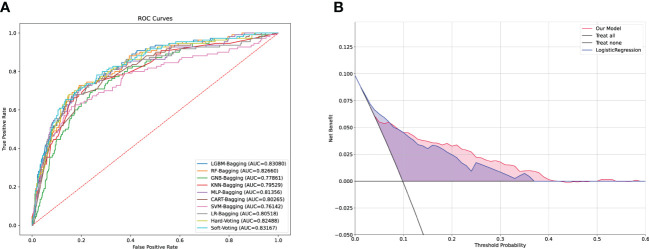
Comparison of the performance among machine learning models and DCA analysis. **(A)** ROC curves of the machine learning in the validation cohort. **(B)** Logistic DCA analysis for two models in the validation cohort. Blue shading (LR model) represents the traditional model only based on logistic regression algorithm, and red shading (stacking model) means the machine model based on ensemble learning strategies and machine learning fusion algorithms in our study. ROC, receiver operating characteristic; DCA, decision curve analysis; AUC, area under curve; LR, logistic regression.

Expectedly, all individual models demonstrated optimal predictive performance in the internal validation set (**Figure 3A**). Additionally, the stacking model exhibited an outstanding predictive performance, with a relatively high AUC of 0.831 and NPV of 97.03%. The AUC, sensitivity, specificity, precision, NPV, FDR, accuracy, AP, F1-sore, and MCC of each model in the internal validation set are listed in **Supplementary Table S2**. An intuitive and concise confusion matrix was also employed to evaluate the performance of the models; the detailed outcomes of model prediction in the internal testing set are shown in (**Supplementary Table S3**).

To further appraise whether our ML-based models performed better than did the classical LR strategy, we fit our data in the LR models. The stacking model had a higher AUC and NPV than the LR model (**Supplementary Table S4**). Considering the clinical implications and with the guidance of the models, we drew a DCA curve by performing a logistic DCA analysis. The corresponding results revealed a favorable net clinical benefit of both the stacking and LR models, although the stacking model had a stronger effect (**Figure 3B**).

### Subgroup analysis

**Figure 4:**
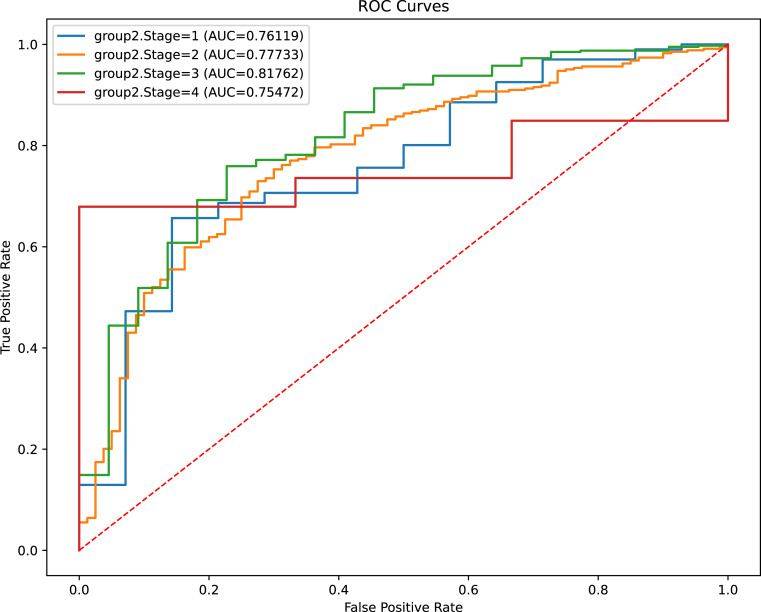
Evaluation of models’ discriminant capability for CRC patients with different clinical stage. ROC, receiver operating characteristic; AUC, area under curve.

To further confirm the comprehensive performance of the stacking model in complicated clinical scenarios, we stratified our cohort into four subgroups according to the AJCC stage. The analysis showed that the stacking model achieved a promising discriminative capacity, irrespective of the tumor stage (**Figure 4**).

### Clinical application

**Figure 5:**
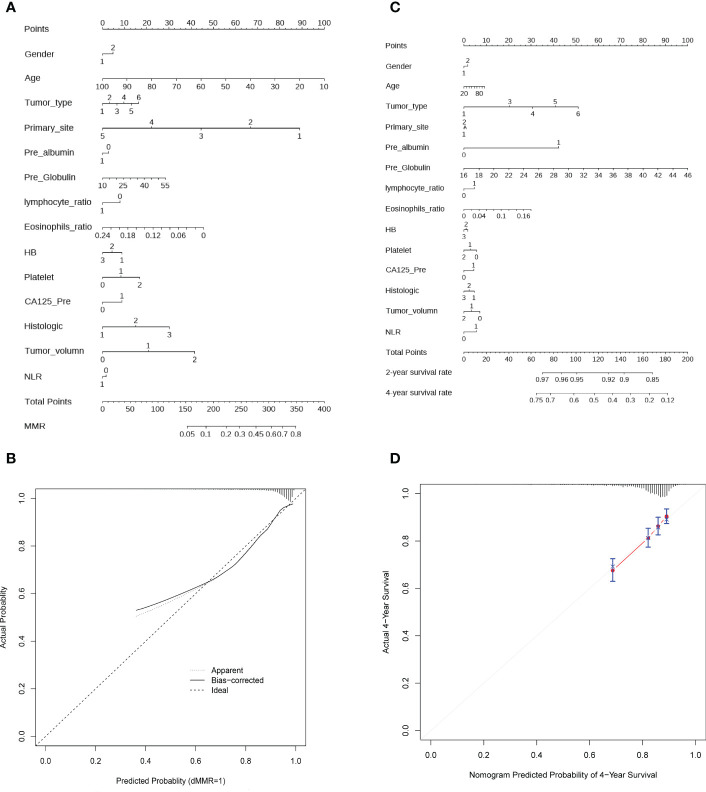
Construction of nomogram and calibration diagram. **(A)** A nomogram to predict the dMMR probability of CRC patients with preoperative routine indexes. In the nomogram, the total points are the sum of individual point for each feature, with larger total points reflecting greater probability of dMMR. **(B)** Calibration curve to evaluate the predictive power of the nomogram. **(C)** Nomogram to predict the 2- and 4- year OS of CRC patient, with higher total points denoting worse prognosis. **(D)** Calibration curve for the estimation of 4-year OS predicted by the nomogram. The diagonal dotted line represents theoretical response of perfect nomogram, the red solid line indicates the performance of nomogram. Abbreviations: HB, hemoglobin; NLR, neutral lymphoid ratio; CA125, carbohydrate antigen 125; dMMR, mismatch repair deficiency.

To facilitate the clinical application of the model, we further developed a concise and accessible nomogram based on 14 crucial features that can be used to accurately assess the MMR status of CRC patients (**Figure 5A**). Thereafter, a calibration curve was adopted to evaluate the predictive power of the nomogram. The calibration curve indicated that the error between the actual and predicted dMMR rates was very small, suggesting that the nomogram possesses a preferable accuracy in predicting dMMR (**Figure 5B**). The features could also well predict the prognosis of CRC patients. Similarly, we constructed a prognostic nomogram and calibration curve, which showed that the predictive power was close to the ideal curve, indicating that the prognostic nomogram has a great predictive capability (**Figure 5C, D**).

## Discussion

dMMR or MSI in CRC patients can serve as a cardinal parameter for clinical decision-making, as it identifies a distinct patient subset with a favorable prognosis, in whom treatment with standard fluorouracil-based chemotherapy has no clinical benefit and in whom ICB treatment might be of remarkable benefit (35). However, in clinical practice, only a portion of CRC patients is tested for dMMR or MSI owing to the associated high costs and dependence on the operator. Therefore, a low-cost, broadly accessible, and robust predictive model for dMMR or MSI that can guide diagnostic and therapeutic strategies is urgently needed.

Recently, an increasing number of researchers harnessed ML as a breakthrough tool for sophisticated medical issues, which has made great clinical contributions, including disease prediction, prognosis evaluation, and new drug development. ML-based models can achieve considerably better accuracy, stability, and interpretability compared with traditional regression models. Thus, we developed and validated an innovative and cost-efficient model to predict dMMR by utilizing eight ML algorithms and combining 14 parameters that are ubiquitously available in clinical practice. Although partial parameters were found to have relatively weak contribution to the model output, they were essential for overall model performance (36). In order to get a higher sensitivity, specificity and prediction power model, we retained all parameters filtered by multiple ML algorithms. Notably, all tests for these parameters were completely standardized and consistent among different regions and hospitals. Thus, the findings could be easily generalized to the complicated clinical environment. The receiver operating characteristic curve analysis showed that both the final fusion model and individual models had a satisfactory performance, while the DCA analysis revealed more net benefits of the fusion model than of the conventional regression model. Most importantly, our model showed a prominent capability in recognizing dMMR in CRC patients regardless of the clinical stage. Ultimately, we constructed a user-friendly nomogram comprising the features of the fusion model that can be used in identifying dMMR in CRC patients and treating them with a personalized strategy.

We established an ML-based model based on adequate clinical data to predict dMMR in CRC patients. Compared with previous analogous studies discerning dMMR or MSI with the combination of histomorphological patterns with features associated with dMMR or MSI from hematoxylin and eosin-stained slides of CRC patients and combination of susceptible genes related to dMMR or MSI with transcriptome data, our study had several considerable strengths. First, we focused on incorporating dMMR and adequate clinical information into our model, which was easily accessible and closely associated with the comprehensive state of the patients. Meanwhile, the detection methods of all features were objective and normalized, which eliminated the variation between hospitals at different levels and between different locations. Notably, all data were derived from preoperative examinations, which could be useful in identifying patients with dMMR early, especially those who could not undergo surgical resection or those who developed distant metastases. Second, all ML-based models achieved a satisfactory discriminative power. More importantly, both the individual and stacking models yielded a crucial NPV (> 0.97), indicating that our model could furnish an additional value as an automatic screening tool to assist in clinical decision-making before confirmatory MMR testing. Specifically, if all patients predicted to have pMMR are excluded from confirmatory detection, this will dramatically decrease the number of patients undergoing MMR testing, yielding substantial test-related labor and cost savings. Third, several previous studies only used a single ML algorithm, while our study utilized ensemble learning strategies and ML fusion algorithms, which generated comprehensive learning with strong robustness and generalizability. Finally, previous studies have suggested that concordance between MMR and MSI analysis is quite high especially in CRC patients (37), so our model may be useful for MSI assessment.

Our findings on the clinicopathological characteristics of dMMR are consistent with previously published data. dMMR was more frequently associated with mucinous adenocarcinoma, poorly differentiated, and mostly located in the right hemicolon (38, 39). Moreover, younger CRC patients were more likely to have dMMR than older patients, which might be related to their higher metabolism rate (40). Notably, beyond these classical risk factors, our research also identified several risk factors particularly relevant for dMMR by adopting multiple ML methods; these factors included a larger tumor volume; lower HB level, albumin level, lymphocyte ratio, and eosinophil ratio; and higher platelet count, globulin level, NLR, and CA125 level. Numerous studies have demonstrated that inflammatory mediators, important promoters of genetic alterations, play an important role in tumor initiation and progression (41). Specifically, inflammatory immune cells, such as macrophages and neutrophils, produce a wide variety of reactive substances, which could directly trigger DNA damage of nonimmune cells to further increase mutation frequency and genomic instability (42). When DNA sustains damage, specific proteins and enzymes are activated to repair such damage (43). During this process, HB and albumin as the primary vectors of nutrients could reduce DNA injury and strengthen the ability to repair DNA damage. Conversely, if the levels of HB and albumin decrease, the capacity to repair DNA damage will distinctly increase (44, 45). These findings coincide with our data on the lower levels of HB and albumin and higher NLR. Accumulating evidence also suggests that platelets play a critical role in angiogenesis modulation, tumor immune microenvironment maintenance, and cancer progression (46). An increased platelet count might be strongly associated with carcinogenesis and prognosis of CRC patients with dMMR (47). Interestingly, eosinophils could have both positive and negative effects: They could both promote tumor progression and exert anti-tumor activities by secreting cytokines (48, 49). Previous studies have also indicated that CA125 could serve as an important diagnostic and prognostic marker in CRC (50). Although several studies reported that these features are closely associated with dMMR in CRC patients, the biological explanations for the underlying mechanisms are not well understood, which is worthy of further exploration.

Because the immune microenvironment and clinicopathological features were varied greatly in different tumors, our model may not be applicable to the assessment of MSI or MMR in other tumors. However, the insights and methods of this study were completely generalized on other tumors. Additionally, this study had several limitations. First, our analysis was conducted at a single medical center; more external test cohorts from different hospitals and regions are of great necessity to enhance the robustness and generalizability of our models. Second, this study had a retrospective design; a large prospective clinical trial is necessary before MMR testing could be routinely performed in clinical practice. Third, although we utilized SHAP values and previous data to interpret the feature performance in our model, further basic science studies are required to determine the underlying mechanisms.

## Conclusions

In conclusion, we successfully confirmed 14 features closely related to dMMR in CRC patients: age, sex, HB level, platelet count, albumin level, globulin level, lymphocyte ratio, eosinophil ratio, NLR, CA125 level, primary site, histologic tumor grade, tumor type, and tumor volume. An innovative and universal fusion model based on multiple ML algorithms was constructed to predict dMMR in CRC patients, which achieved a satisfactory predictive performance. A user-friendly nomogram was also adopted, which yielded benefits and demonstrated prospects for clinical application.

## Data availability statement

The original contributions presented in the study are included in the article/**Supplementary Material**. Further inquiries can be directed to the corresponding authors.

## Ethics statement

Written informed consent was not obtained from the individual(s) for the publication of any potentially identifiable images or data included in this article.

## Author contributions

JL and JZ designed the study. DX, RC, and YJ contributed to the conception of the study and completed the manuscript together. SW, ZL and XC contributed significantly to statistical analysis and manuscript preparation. XF and JZ helped perform the analysis with constructive discussions. All authors contributed to the article and approved the submitted version.
