# Supplementary material for: Application of machine learning in the prediction of deficient mismatch repair in patients with colorectal cancer based on routine preoperative characterization
Source: Front Oncol. 2022 Dec 22;12:1049305. doi: 10.3389/fonc.2022.1049305 (PMC9814116; doi:10.3389/fonc.2022.1049305)
Supplement: Supplementary file 1 [file DataSheet_1.docx]

Table S1 Significance of clinical features in ML models.

| **Model** | **Gender** | **Age** | **Tumor type** | **Primary site** | **Albumin** | **Globulin** | **Lymphocyte.**  **ratio** | **Eosinophils ratio** | **HB** | **Platelet** | **CA125** | **Histologic**  **grade** | **Tumor volume** | **NLR** |
| --- | --- | --- | --- | --- | --- | --- | --- | --- | --- | --- | --- | --- | --- | --- |
| LGBM | 0.1563 | 0.4375 | 0.0000 | 0.3438 | 0.1250 | 0.0625 | 0.4063 | 0.1563 | 0.6250 | 0.1250 | 0.3438 | 1.0000 | 0.7813 | 0.2187 |
| RF | 0.2703 | 0.2432 | 0.0541 | 0.4054 | 0.1892 | 0.1351 | 0.3243 | 0.5946 | 0.4324 | 0.3514 | 0.2973 | 0.6216 | 1.0000 | 0.0000 |
| GNB | 0.3976 | 0.4819 | 0.3976 | 0.4096 | 0.1446 | 0.2169 | 0.2771 | 0.5904 | 0.0000 | 0.4337 | 0.2048 | 0.5301 | 1.0000 | 0.1687 |
| KNN | 0.4063 | 0.6563 | 0.2813 | 0.3125 | 0.3438 | 0.0000 | 0.1875 | 0.4688 | 1.0000 | 0.5625 | 0.7500 | 0.4688 | 0.2188 | 0.2500 |
| MLP | 0.2020 | 0.4286 | 0.0000 | 1.0000 | 0.0246 | 0.1527 | 0.3251 | 0.0985 | 0.3005 | 0.0591 | 0.1182 | 0.0345 | 0.1921 | 0.1034 |
| CART | 0.0000 | 0.1167 | 0.0000 | 1.0000 | 0.0077 | 0.0042 | 0.0000 | 0.0000 | 0.0000 | 0.0004 | 0.0024 | 0.0032 | 0.3338 | 0.0000 |
| SVM | 0.2922 | 0.4675 | 0.4299 | 0.1802 | 0.9512 | 1.0000 | 0.3073 | 0.0000 | 0.8894 | 0.6011 | 0.9459 | 0.5901 | 0.8154 | 0.7467 |
| LR | 0.8500 | 0.4500 | 0.3250 | 0.0000 | 0.9000 | 0.5750 | 0.7750 | 0.8250 | 0.7000 | 0.8250 | 0.9000 | 1.0000 | 0.4250 | 0.7750 |
| Stacking | 0.3218 | 0.4102 | 0.1860 | 0.4564 | 0.3358 | 0.2683 | 0.3253 | 0.3417 | 0.4934 | 0.3698 | 0.4453 | 0.5310 | 0.5958 | 0.2828 |

Abbreviations: ML: Machine learning; CA125, Carbohydrate antigen 125; HB, hemoglobin; NLR, Neutral lymphoid ratio; LGBM, Light Gradient Boosting Machine; RF, Random Forest; GNB, Gaussian Naive Bayesian; KNN, K-Nearest Neighbor; MLP, Multilayer Perceptron; CART, Classification and Regression Trees; SVM, Support Vector Machine; LR, Logistic Regression.

Table S2: Performance of developed models in validation dataset.

| **Models** | **AUC** | **Sensitivity** | **Specificity** | **Precision** | **NPV** | **FDR** | **Accuracy** | **AP** | **F1** | **MCC** |
| --- | --- | --- | --- | --- | --- | --- | --- | --- | --- | --- |
| LGBM | 0.8308 | 0.8000 | 0.6782 | 0.2131 | 0.9689 | 0.7869 | 0.6902 | 0.3713 | 0.3365 | 0.2950 |
| RF | 0.8266 | 0.7636 | 0.7030 | 0.2188 | 0.9647 | 0.7812 | 0.7089 | 0.3756 | 0.3401 | 0.2926 |
| GNB | 0.7786 | 0.7364 | 0.7020 | 0.2120 | 0.9607 | 0.7880 | 0.7054 | 0.2554 | 0.3293 | 0.2752 |
| KNN | 0.7953 | 0.7545 | 0.7168 | 0.2249 | 0.9640 | 0.7751 | 0.7205 | 0.3554 | 0.3466 | 0.2985 |
| MLP | 0.8136 | 0.7727 | 0.7050 | 0.2219 | 0.9661 | 0.7781 | 0.7116 | 0.3730 | 0.3448 | 0.2997 |
| CART | 0.8026 | 0.7455 | 0.7158 | 0.2222 | 0.9627 | 0.7778 | 0.7188 | 0.3016 | 0.3424 | 0.2921 |
| SVM | 0.7614 | 0.7273 | 0.6535 | 0.1860 | 0.9565 | 0.8140 | 0.6607 | 0.3296 | 0.2963 | 0.2330 |
| LR | 0.8052 | 0.7636 | 0.7257 | 0.2327 | 0.9657 | 0.7673 | 0.7295 | 0.3235 | 0.3567 | 0.3116 |
| Stacking | 0.8317 | 0.8000 | 0.7109 | 0.2316 | 0.9703 | 0.7684 | 0.7196 | 0.3827 | 0.3592 | 0.3211 |

AUC, area under curve; NPV, negative predictive value; FDR, false discovery rate; AP, average precision; MCC, Matthews correlation coefficient; LGBM, Light Gradient Boosting Machine; RF, Random Forest; GNB, Gaussian Naive Bayesian; KNN, K-Nearest Neighbor; MLP, Multilayer Perceptron; CART, Classification and Regression Trees; SVM, Support Vector Machine; LR, Logistic Regression

| Confusion matrix | Inner validation |  |  |
| --- | --- | --- | --- |
|  | Actual | Prediction |  |
|  |  | pMMR | dMMR |
| LGBM | pMMR | 685 | 325 |
|  | dMMR | 22 | 88 |
| RF | pMMR | 710 | 300 |
|  | dMMR | 26 | 84 |
| GNB | pMMR | 709 | 301 |
|  | dMMR | 29 | 81 |
| KNN | pMMR | 724 | 286 |
|  | dMMR | 27 | 83 |
| MLP | pMMR | 712 | 298 |
|  | dMMR | 25 | 85 |
| CART | pMMR | 723 | 287 |
|  | dMMR | 28 | 82 |
| SVM | pMMR | 660 | 350 |
|  | dMMR | 30 | 80 |
| LR | pMMR | 733 | 277 |
|  | dMMR | 26 | 84 |
| Stacking | pMMR | 718 | 292 |
|  | dMMR | 22 | 88 |

**Table S3.** Confusion matrix of developed ML model

ML Machine Learning, dMMR Mismatch repair deficiency, pMMR Mismatch repair proficiency, LGBM Light Gradient Boosting Decision, RF Random Forest, GNB Gaussian Naive Bayesian, KNN K-Nearest Neighbor, MLP Multilayer Perceptron, CART Classification and Regression Trees, SVM Support Vector Machine, LR Logistic Regression.

Table S4 Comparison of ML algorithms and logistic regression algorithm.

| **Model** | **Formula** | **AUC** |
| --- | --- | --- |
| Stack-bagging (ML algorithm) | - | **0.832** |
| Logistic regression with  data normalization by ML algorithm | e^(2.52302451-0.02647073*Gender+0.32546871*Age+0.00441319*Tumor_type  +0.71943147*Primary_site+0.1240955*Pre_albumin-0.02461756*Pre_Globulin+0.0053605*lymphocyte_ratio+0.02031354*Eosinophils_ratio+0.1377018*HB-0.02398963*Platelet-0.00155924*CA125_Pre-0.22249936*Histologic_grade-0.42587652*Tumor_volumn+0.00094851*NLR) | **0.807** |
| Logistic regression algorithm | e^(0.32926274-0.04746376*Gender+0.0242436*Age+0.00905698*Tumor_type  +1.44335578*Primary_site+0.73979414*Pre_albumin+0.01727405*Pre_Globulin+0.20875081*lymphocyte_ratio+0.02565158*Eosinophils_ratio+0.00071247*HB-0.00915419*Platelet-0.05680438*CA125_Pre-0.41082887*Histologic_grade-0.82216006*Tumor_volumn-0.00117396*NLR) | **0.805** |

ML, machine learning; CA125, carbohydrate antigen 125; NLR, neutral lymphoid ratio.
